# Multi-target neuroprotective mechanisms of epigallocatechin-3-gallate (EGCG) in stroke: from molecular pathways to nanomedicine delivery and clinical translation

**DOI:** 10.3389/fphar.2026.1925262

**Published:** 2026-09-22

**Authors:** Yifan Yuan, Cuimei Xie, Yating Huang, Jun Xi, Peipei Wang, Zhiying Chen, Xiangbin Wu, Yijun Suo, Xiaoping Yin, Bing Bao

**Affiliations:** 1 Jiujiang University Medical College, Jiujiang, China; 2 Department of Neurology, Jiujiang University Affiliated Hospital, Jiujiang, China

**Keywords:** clinical translation, EGCG, nanotechnology, neuroprotection, stroke

## Abstract

Stroke ranks among the leading global causes of mortality and permanent disability. Its secondary brain injury arises from multiple interrelated pathological cascades, including oxidative stress, neuroinflammation, disrupted autophagy-apoptosis balance, excitotoxicity, iron dyshomeostasis, and blood–brain barrier (BBB) dysfunction. Although intravenous thrombolysis and mechanical thrombectomy have greatly improved the efficacy of acute reperfusion therapy, effective neuroprotective regimens for secondary brain injury remain scarce. Epigallocatechin-3-gallate (EGCG), the most abundant and biologically potent natural catechin extracted from green tea, has attracted extensive research attention in recent years due to its multi-target pharmacological effects. Accumulating evidence demonstrates that EGCG coordinately modulates oxidative stress, neuroinflammation, autophagy-apoptosis homeostasis, excitotoxicity, iron metabolism, and BBB and neurovascular unit (NVU) function to alleviate secondary brain damage and facilitate neurological functional recovery. In addition, EGCG exerts potential adjunct antiplatelet effects *via* inhibiting platelet activation and thrombosis in ischemic stroke models. Nevertheless, the clinical translation of free EGCG is hindered by poor chemical stability, low systemic bioavailability, limited exposure in cerebral tissue, and insufficient BBB penetration. In recent years, nanodelivery platforms such as liposomes, polymeric nanoparticles, exosomes, and metal–organic frameworks (MOFs) have been proven to markedly enhance EGCG stability, brain-targeted delivery efficiency, and overall bioavailability. These nanocarriers exert enhanced neuroprotective effects and confer synergistic therapeutic benefits when combined with standard agents such as nimodipine in experimental stroke models, offering novel strategies to overcome the translational bottlenecks of free EGCG. This review systematically summarizes research advances in EGCG’s multi-target neuroprotective mechanisms, nanocarrier delivery systems, and translational prospects, focusing on analyzing the strengths and limitations of existing evidence and identifying future research directions. Future investigations should prioritize optimizing brain-targeted delivery platforms, standardizing pharmacokinetic evaluation, unifying therapeutic dose specifications, and conducting high-quality randomized controlled trials to accelerate the clinical transformation of EGCG nanoformulations for precision stroke therapy.

## 1 Introduction

Stroke is the world’s second leading cause of death and the primary contributor to long-term disability, representing a severe public health crisis with a continuously rising global disease burden (GBD 2021 Stroke Risk Factor Collaborators, 2024). According to the 2021 Global Burden of Disease (GBD) data, approximately 11.9 million new stroke cases, 7.1 million stroke-related deaths, and over 160 million disability-adjusted life years (DALYs) were recorded worldwide in 2021, with disproportionately severe disease burdens observed in low- and middle-income nations (GBD 2021 Stroke Risk Factor Collaborators, 2024). Etiologically, stroke is classified into ischemic stroke (IS) and hemorrhagic stroke (HS). While intravenous thrombolysis, mechanical thrombectomy, and neurocritical care have remarkably improved the prognosis of acute stroke patients, most survivors suffer persistent neurological deficits due to narrow therapeutic time windows and restricted patient eligibility alongside reperfusion injury. Therefore, developing novel neuroprotective interventions to mitigate secondary brain injury and restore neurological function remains a core research priority in cerebrovascular disease (Powers et al., 2019; Greenberg et al., 2022).

Mounting evidence reveals that a single pathological event does not trigger post-stroke secondary brain injury. Still, it results from the cascading amplification of mutually interactive pathological processes, including oxidative stress, neuroinflammation, autophagy-apoptosis imbalance, excitotoxicity, iron dyshomeostasis, BBB breakdown, and NVU dysfunction (Chamorro et al., 2016). These pathological alterations interweave throughout stroke onset, progression, and neurological recovery, which explains the unsatisfactory clinical outcomes of single-target neuroprotective agents. Natural bioactive compounds capable of coordinately regulating multiple signaling pathways have thus become a focal point of stroke neuroprotection research (Khan and Mukhtar, 2007).

Epigallocatechin-3-gallate (EGCG) is the most abundant and biologically active catechin in green tea, and also one of the most widely investigated natural polyphenols. A large body of preclinical studies has demonstrated that EGCG concurrently regulates oxidative stress, neuroinflammation, autophagy-apoptosis dysregulation, excitotoxicity, iron imbalance, and BBB damage, while promoting neurogenesis and angiogenesis to alleviate multi-layered secondary brain injury and improve functional recovery, thereby showing prominent neuroprotective potential (Khan and Mukhtar, 2007; Singh et al., 2011; Iadecola et al., 2020). However, the clinical application of free EGCG faces a critical bottleneck: conventional administration fails to achieve and sustain therapeutically effective cerebral concentrations, creating a substantial gap between the effective doses observed in preclinical assays and the safe exposure levels achievable in human subjects (Lin et al., 2007; Pervin et al., 2017).

Advancements in nanodelivery technology have offered viable solutions to overcome the above limitations. Nanocarriers, including liposomes, polymeric nanoparticles, exosomes, and metal–organic frameworks (MOFs), can improve EGCG chemical stability, prolong systemic circulation time, enhance BBB permeability and cerebral-targeted accumulation, enable sustained cargo release, and support combinatorial therapy, thereby amplifying its neuroprotective efficacy (Niu et al., 2022; Bakun et al., 2023). With ongoing innovations in brain-targeted and stimuli-responsive nanoplatforms, EGCG nanoformulations hold broad potential to optimize pharmacokinetic profiles, elevate cerebral drug exposure, and advance clinical translation.

Existing review articles separately elaborate the pharmacological activity of EGCG, neuroprotective effects of natural polyphenols, or the development of individual nanocarriers. In contrast, no comprehensive review integrates EGCG’s multi-target signaling mechanisms, nanodelivery strategies, and translational evidence. Against this backdrop, the present review systematically summarizes EGCG’s neuroprotective effects across antioxidant, anti-inflammatory, autophagy- and apoptosis-regulatory, anti-excitotoxic, iron-homeostatic, BBB/NVU-protective, neurorestorative, and antiplatelet aspects, centered on core stroke pathological mechanisms. It further highlights cutting-edge research on EGCG nanocarriers and their applications in stroke models. In conjunction with available clinical data, this paper discusses translational prospects, unresolved obstacles, and future research directions, aiming to provide theoretical guidance for the rational design of EGCG nanoformulations and precision stroke therapy.

Literature retrieval was performed across PubMed, Web of Science, Scopus, and Google Scholar, covering publications from database inception to July 2026. Search terms included epigallocatechin-3-gallate, EGCG, stroke, ischemic stroke, cerebral ischemia, intracerebral hemorrhage, subarachnoid hemorrhage, neuroprotection, blood–brain barrier, ferroptosis, autophagy, nanoparticle, liposome, exosome, and metal–organic framework. Original studies directly evaluating EGCG efficacy in stroke cell lines, animal models, or human patients were prioritized for inclusion. Non-stroke research articles were only incorporated if they provided direct mechanistic or formulation evidence relevant to stroke, with their indirect nature explicitly annotated in the main text.

## 2 Multi-target neuroprotective mechanisms of EGCG

Post-stroke secondary brain injury involves interconnected pathological events including oxidative stress, neuroinflammation, autophagy-apoptosis imbalance, excitotoxicity, iron dyshomeostasis, BBB/NVU damage, and impaired neural repair. Cumulative studies confirm that EGCG orchestrates a multi-pathway regulatory network covering acute neuroprotection and chronic tissue remodeling, and inhibits platelet activation in ischemic stroke models. Figure 1 summarizes the overall neuroprotective signaling cascade of EGCG and the crosstalk between distinct pathways, with detailed elaborations presented in subsequent subsections.

**FIGURE 1:**
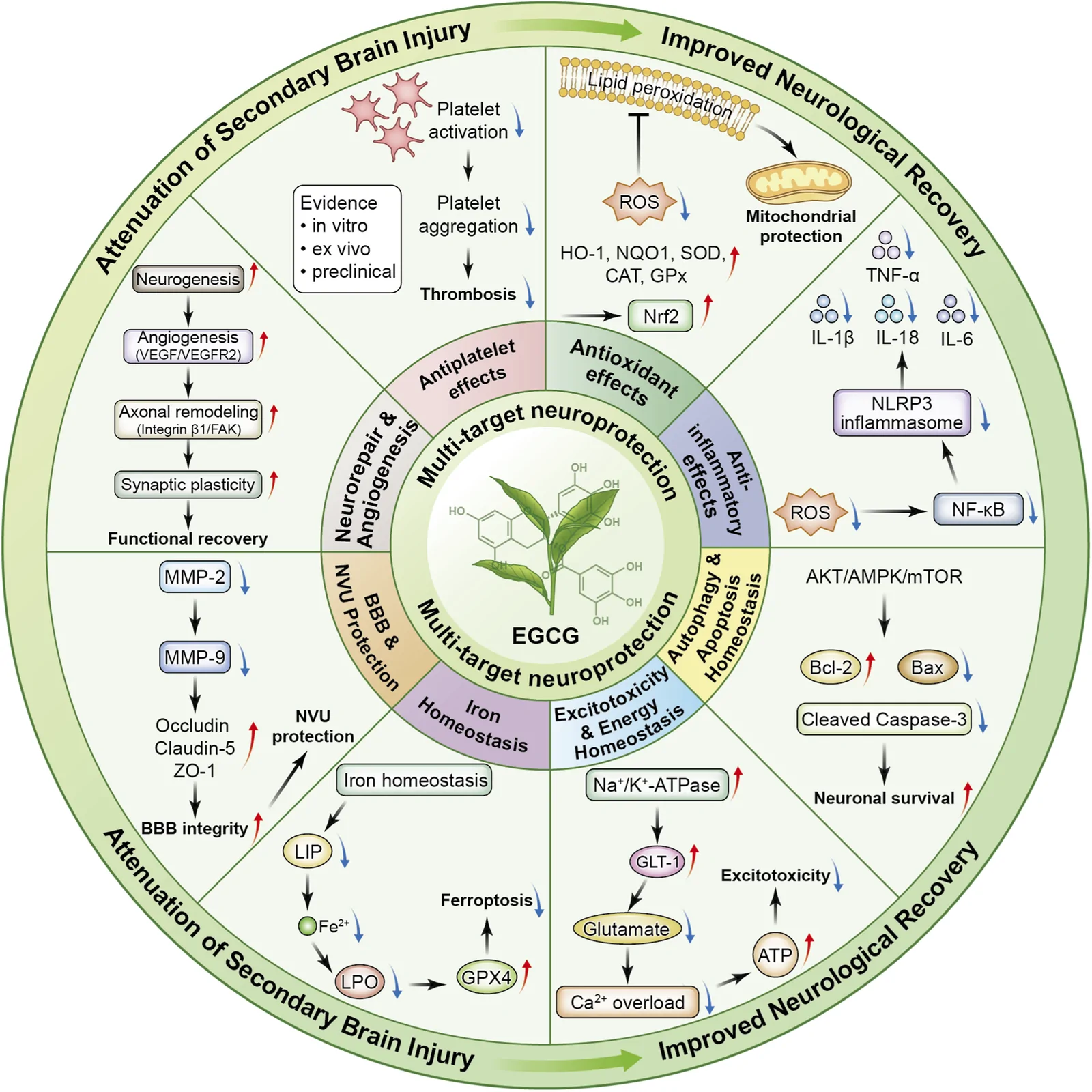
Multi-target neuroprotective mechanisms of epigallocatechin-3-gallate (EGCG) in stroke.

### 2.1 Synergistic antioxidant and anti-inflammatory effects of EGCG

Following stroke onset, cerebral ischemia and reperfusion rapidly trigger massive reactive oxygen species (ROS) overproduction, inducing oxidative stress, mitochondrial dysfunction, and BBB injury, which further activate neuroinflammation to form a self-amplifying vicious cycle that exacerbates secondary brain damage. Concurrent suppression of oxidative stress and neuroinflammation has thus become a core therapeutic strategy for stroke neuroprotection. As the most abundant and potent catechin in green tea, EGCG ranks among the most extensively studied natural polyphenols. Numerous investigations have demonstrated that EGCG exerts multi-target neuroprotection through coordinated modulation of multiple signaling cascades (Liu et al., 2022; Wang et al., 2025).

The Keap1/Nrf2/HO-1 axis constitutes a pivotal molecular pathway mediating EGCG’s antioxidant activity. Under oxidative stress, EGCG facilitates Nrf2 dissociation from the Keap1 complex and promotes Nrf2 nuclear translocation to bind the antioxidant response element (ARE), initiating transcription of downstream antioxidant genes encoding heme oxygenase-1 (HO-1), NAD(P)H quinone oxidoreductase 1 (NQO1), superoxide dismutase (SOD), catalase (CAT), and glutathione peroxidase (GPx). These enzymes strengthen endogenous antioxidant capacity, eliminate excess ROS, mitigate lipid peroxidation, stabilize mitochondrial redox homeostasis, and ultimately reduce neuronal loss after cerebral ischemia (Xu et al., 2021; Zhou et al., 2021).

Oxidative stress and neuroinflammation do not operate independently but form a tightly coupled positive feedback loop during stroke pathogenesis. Excessive ROS not only inflict direct oxidative cellular damage but also act as upstream signaling molecules to activate NF-κB, driving robust secretion of pro-inflammatory mediators including tumor necrosis factor-α (TNF-α), interleukin-1β (IL-1β), interleukin-6 (IL-6), inducible nitric oxide synthase (iNOS), and cyclooxygenase-2 (COX-2), which further amplify neuroinflammatory lesions. Correspondingly, EGCG suppresses NF-κB activation and reduces pro-inflammatory cytokine release, thereby remodeling the inflammatory microenvironment post-stroke (Liu et al., 2022; Wang et al., 2025).

Recent research identifies the NLRP3 inflammasome as a critical molecular hub linking oxidative stress and neuroinflammation. ROS-mediated TXNIP/NLRP3 complex assembly activates caspase-1, thereby facilitating the maturation and secretion of IL-1β and IL-18, amplifying sterile neuroinflammation after stroke. Existing evidence indicates that EGCG indirectly inhibits NLRP3 inflammasome priming and assembly by reducing ROS generation, blunting NF-κB signaling, and restoring cellular redox balance, thereby alleviating neuroinflammation and breaking the pathological cycle between oxidation and inflammation (Liu et al., 2022; Wang et al., 2022b).

Notably, the effective dose, route of administration, and intervention window for EGCG vary substantially across cellular and animal models, precluding the establishment of a universal optimal therapeutic dose. Interpretations of EGCG’s antioxidant and anti-inflammatory efficacy must account for stroke subtype, injury severity, treatment timing, and experimental platforms, and avoid drawing absolute dose-specific conclusions from single investigations. Collectively, these studies demonstrate that EGCG coordinately modulates the Keap1/Nrf2/HO-1, NF-κB, and ROS/NLRP3 signaling pathways to suppress oxidative stress and neuroinflammation simultaneously. Nevertheless, persistent oxidative and inflammatory insults further disrupt intracellular quality-control systems, in which autophagy-apoptosis crosstalk determines neuronal survival or death.

### 2.2 Multi-layered modulation of autophagy-apoptosis crosstalk by EGCG

Beyond oxidative stress and neuroinflammation, dysregulated intracellular quality surveillance represents another pivotal driver of neuronal damage post-stroke. Ischemia and reperfusion aberrantly activate autophagy and apoptosis; the two processes share overlapping signaling molecules and mutually regulate each other to govern neuronal fate. Restoring autophagic homeostasis and maintaining dynamic equilibrium between autophagy and apoptosis have therefore emerged as essential neuroprotective strategies. Cumulative evidence indicates that EGCG harmonizes autophagy-apoptotic networks to elicit multi-target neuroprotection (Shi et al., 2021; Wang et al., 2022a).

The AKT/AMPK/mTOR cascade is central to EGCG-mediated normalization of pathological autophagy. Cerebral ischemia induces sustained AMPK activation and mTOR suppression, thereby elevating Beclin1 expression and LC3-II accumulation, triggering excessive, detrimental autophagy. In MCAO/R mice and OGD/R HT22 neuronal models, Wang et al. reported that EGCG restores phosphorylated AKT and mTOR levels, modulates AMPK activity, downregulates Beclin1 and the LC3II/LC3I ratio, and reverses aberrant p62 aggregation, simultaneously reducing infarct volume and neuronal apoptosis. Pretreatment with pathway inhibitors markedly abrogates EGCG’s protective effects, thereby validating the functional involvement of this signaling axis (Wang et al., 2022a; 2025).

While rectifying aberrant autophagy, EGCG further coordinates functional crosstalk between autophagy and apoptosis. The Beclin1–Bcl-2 complex acts as a master switch governing the balance of the 2 cell death programs. Under ischemic conditions, Beclin1 dissociates from Bcl-2 to initiate hyperactive autophagy; concurrently, an elevated Bax/Bcl-2 ratio and caspase-3 cleavage trigger mitochondrial-mediated apoptosis. Studies reveal that EGCG upregulates anti-apoptotic Bcl-2, downregulates pro-apoptotic Bax and cleaved caspase-3, suppresses cytochrome c efflux, and tunes Beclin1-dependent autophagic activity to sustain autophagy-apoptosis equilibrium. In addition to targeting the Beclin1–Bcl-2 axis, EGCG activates the PI3K/Akt survival pathway to reinforce anti-apoptotic defenses, thereby generating synergistic, multi-layered cytoprotection. This holistic remodeling of cell-fate signaling preserves neuronal viability and strengthens EGCG’s efficacy against post-stroke secondary injury (Shi et al., 2021; Liu et al., 2022; Han et al., 2025).

In summary, EGCG’s regulation of post-stroke cellular homeostasis relies on coordinated remodeling of autophagic, apoptotic, and cell-survival cascades rather than on single-pathway intervention. However, merely rebalancing cell death signaling cannot fully arrest neuronal degeneration, as excitotoxicity and impaired energy metabolism continuously propagate pathological damage.

### 2.3 Modulation of excitotoxicity and neuronal energy homeostasis by EGCG

Excitotoxicity and disrupted energy metabolism constitute core pathological drivers of irreversible neuronal injury during post-stroke secondary damage. Cerebral ischemia abruptly interrupts oxygen and glucose supply, diminishing mitochondrial ATP production and impairing Na^+^/K^+^-ATPase function. This weakens glutamate transporter 1 (GLT-1)-mediated glutamate clearance, leading to extracellular glutamate accumulation and the onset of excitotoxicity. Sustained overactivation of glutamate receptors subsequently induces intracellular Ca^2+^ overload, exacerbates mitochondrial dysfunction, and establishes a self-perpetuating cycle of energy depletion, ionic imbalance, and neuronal death. Multiple lines of evidence demonstrate that EGCG interrupts this injury cascade at multiple nodes, thereby sustaining neuronal energy homeostasis post-stroke (Pivovarova and Andrews, 2010; Peng et al., 2019; Belov Kirdajova et al., 2020; Zhu et al., 2022).

Restoration of ionic equilibrium represents a primary mechanism through which EGCG mitigates excitotoxicity. In MCAO reperfusion rats and OGD/R cellular models, EGCG restores Na^+^/K^+^-ATPase activity, upregulates astrocytic Na^+^/K^+^ α2 subunit (NKAα2) and GLT-1 expression, and prevents ischemia-induced dissociation of the NKAα2–GLT-1 complex, enhancing glutamate clearance, reducing infarct volume, and ameliorating neurological deficits. These observations suggest that EGCG not only restores ionic balance in neuronal membranes but also preserves neuron-astrocyte glutamate cycling, thereby mitigating persistent excitotoxic brain lesions (Liu et al., 2025).

Beyond facilitating glutamate clearance, EGCG sustains intracellular Ca^2+^ homeostasis. In a rat middle cerebral artery occlusion model, Park et al. demonstrated that EGCG reverses ischemia-mediated downregulation of hippocalcin (a neuronal Ca^2+^ sensor protein), alleviates intracellular Ca^2+^ overload, concurrently increases Bcl-2 expression and reduces Bax and cleaved caspase-3 levels, thereby attenuating glutamate-triggered neuronal injury. This indicates that EGCG’s modulation of Ca^2+^ homeostasis synergistically suppresses mitochondrial-dependent apoptosis alongside excitotoxic injury (Park et al., 2024).

Furthermore, EGCG ameliorates post-ischemic energy dysfunction. In a rat cardiac arrest/cardiopulmonary resuscitation global cerebral ischemia model, Qin et al. observed elevated cerebral ATP levels following EGCG administration, implying its capacity to preserve mitochondrial bioenergetics. In experimental subarachnoid hemorrhage (SAH) models, EGCG mitigates oxyhemoglobin-induced Ca^2+^ influx and mitochondrial membrane potential collapse, corrects disrupted mitochondrial fission-fusion dynamics, and reduces mitochondrial fragmentation and cytochrome c release, thereby alleviating neuronal loss and further verifying its mitochondrial protective activity (Chen et al., 2018; Qin et al., 2019).

Collectively, these data illustrate that EGCG counteracts excitotoxicity *via* regulating ionic balance, glutamate turnover, and mitochondrial function. Nevertheless, all supporting evidence originates from preclinical cellular and animal studies; direct human clinical data validating EGCG’s regulation of the NKAα2–GLT-1 complex, Ca^2+^ signaling, and cerebral energy metabolism remain scarce. Even partial restoration of metabolic function fails to eliminate iron-dependent oxidative damage, rendering iron homeostasis a burgeoning focus of contemporary stroke neuroprotection research.

### 2.4 Regulation of iron homeostasis by EGCG

Dysregulated iron homeostasis has been identified as a critical pathological bridge linking oxidative stress, lipid peroxidation, and ferroptosis, serving as a major contributor to post-stroke secondary brain injury. In hemorrhagic stroke, erythrocyte lysis releases abundant hemoglobin and heme, which are degraded by heme oxygenase-1 (HO-1) to generate massive free Fe^2+^. In ischemic reperfusion injury, inflammation, acidosis, and BBB breakdown also disrupt iron trafficking and induce intracellular accumulation of labile iron. Excess free ferrous iron initiates Fenton chemistry, triggering lipid peroxidation and mitochondrial dysfunction, thereby activating ferroptotic cell death. Remodeling of cerebral iron homeostasis has thus emerged as a promising therapeutic target for stroke (Zhang H. et al., 2021; Sun et al., 2022).

Restricting abnormal expansion of the labile iron pool (LIP) is central to EGCG-mediated maintenance of iron homeostasis. The polyphenolic hydroxyl moieties of EGCG chelate free Fe^2+^/Fe^3+^ to limit substrates for the Fenton reaction; concurrently, EGCG modulates HO-1-dependent iron metabolic reprogramming and suppresses NCOA4-mediated ferritinophagy to block aberrant mobilization of stored iron, thereby alleviating iron overload, restoring mitochondrial function, and inhibiting ferroptosis. Studies in SA and SAH models confirm that EGCG rectifies cerebral iron imbalance, reduces iron deposition and lipid peroxidation, and promotes neurological recovery. It should be noted that direct evidence linking HO-1-driven iron remodeling is predominantly derived from EGCG nanoformulation studies and cannot be fully extrapolated to the intrinsic pharmacological effects of free EGCG (Lin et al., 2022; Huang et al., 2024; Wang et al., 2025).

Beyond limiting iron accumulation, EGCG reinforces cellular anti-ferroptotic defense systems. Preclinical data reveal that EGCG amplifies Nrf2-mediated antioxidant signaling to sustain the SLC7A11–GSH–GPX4 anti-lipid peroxidation axis, lowers intracellular Fe^2+^ and malondialdehyde (MDA) levels, and restores glutathione (GSH) reserves to suppress iron-dependent lipid peroxidation. However, direct evidence that EGCG upregulates GPX4 remains limited; its anti-ferroptotic efficacy arises from the combined actions of iron chelation, redox normalization, and activated antioxidant signaling rather than from GPX4 overexpression alone (Hao et al., 2024; Wang et al., 2025).

Notably, HO-1 exerts divergent context-dependent effects across stroke subtypes. Moderate Nrf2/HO-1 activation enhances antioxidant defense in ischemic reperfusion lesions, whereas excessive HO-1 induction following intracerebral hemorrhage (ICH) or SAH drives sustained Fe^2+^ release to exacerbate iron overload and lipid peroxidation. Rather than simply activating or inhibiting HO-1, EGCG dynamically tunes its activity according to the pathological context: it exerts antioxidant protection under ischemic conditions while restraining HO-1-mediated iron efflux in hemorrhagic lesions, thereby bidirectionally maintaining cerebral iron homeostasis (Hao et al., 2024; Huang et al., 2024).

These investigations expand the mechanistic spectrum of EGCG’s neuroprotective effects, demonstrating that beyond alleviating oxidative stress, EGCG remodels cellular iron metabolism and inhibits ferroptosis. However, supporting evidence is predominantly preclinical, with many studies relying on nanocarriers to enhance intracerebral EGCG accumulation, necessitating further validation of its translational potential in humans. Moreover, disrupted iron homeostasis secondarily damages vascular endothelium and impairs BBB/NVU function, highlighting iron homeostasis as a foundational prerequisite for stable cerebral microenvironment function.

### 2.5 EGCG-mediated protection against BBB disruption and NVU dysfunction

Impaired BBB function constitutes a fundamental pathological substrate of post-stroke secondary brain injury, acting as a key driver of inflammatory cell infiltration, cerebral edema, and progressive neurological deterioration. Ischemia and reperfusion persistently activate oxidative and inflammatory signaling, leading to aberrant overexpression of matrix metalloproteinases (MMPs), which degrade vascular basement membranes and disrupt tight junction proteins, ultimately increasing BBB permeability and disrupting NVU equilibrium. Maintaining BBB structural and functional integrity has therefore become a key therapeutic objective for stroke neuroprotection.

Inhibition of MMP-mediated vascular barrier degradation is a primary BBB-protective mechanism of EGCG. In rat thromboembolic cerebral ischemia models, delayed recombinant tissue plasminogen activator (rt-PA) administration markedly increases BBB permeability and exacerbates cerebral edema; co-administration of EGCG attenuates BBB leakage, shrinks infarct volume, and improves neurological outcomes, an effect primarily attributed to EGCG-mediated suppression of MMP-induced degradation of basement and tight junction proteins (You, 2016).

Beyond inhibiting MMP activation, EGCG directly maintains the structural integrity of cerebral microvascular endothelial cells. In human cerebral microvascular endothelial cell (hCMEC) models, EGCG reverses lipopolysaccharide (LPS)-evoked downregulation of Occludin and Claudin-5, preserves continuous membranous localization of Claudin-5 and ZO-1 at intercellular junctions, elevates transendothelial electrical resistance (TEER), and reduces endothelial monolayer permeability. Stabilization of tight junction complexes forms the molecular basis of EGCG’s endothelial barrier protection (Li et al., 2012).

Crucially, BBB protection is not limited to endothelial cells but encompasses the holistic maintenance of NVU function. Cumulative evidence indicates that EGCG activates the Nrf2/ARE signaling cascade to upregulate antioxidant molecules including HO-1, eliminate ROS, mitigate oxidative endothelial damage, and sustain functional coupling among neurons, glia, and cerebral microvessels to restore NVU homeostasis (Han et al., 2014).

A small number of preliminary clinical studies provide exploratory support for the mechanisms outlined above. One randomized, double-blind, placebo-controlled trial evaluated adjuvant EGCG therapy in ischemic stroke patients receiving rt-PA. The results indicated that EGCG administration was associated with improved NIHSS scores and reduced plasma concentrations of MMP-2 and MMP-9 in the subgroup receiving delayed treatment (Pervin et al., 2017).

It is critical to distinguish BBB-protective activity from intrinsic BBB penetrance of EGCG. Although preclinical pharmacokinetic assays detect EGCG in discrete cerebral regions and demonstrate cumulative intracerebral exposure after repeated dosing, absolute cerebral tissue concentrations remain low and vary with route of administration, metabolic stability, sampling time, and detection modalities (Lin et al., 2007; Pervin et al., 2017). Current research does not confirm whether measured intracerebral concentrations reach the therapeutically effective thresholds identified in in vitro mechanistic assays. Insufficient cerebral delivery remains a major translational barrier and the primary impetus for developing brain-targeted nanodelivery platforms.

In summary, EGCG’s BBB-protective effects stem from coordinated modulation of oxidative stress, inflammation, and NVU homeostasis rather than isolated stabilization of tight junctions. This integrated regulatory function limits acute secondary injury and establishes a permissive microenvironment for cerebral remodeling during the recovery phase.

### 2.6 EGCG promotes post-stroke neural repair: From neurogenesis to neural network remodeling

Unlike acute-phase neuroprotective interventions that block injury cascades, long-term neurological recovery post-stroke relies on cerebral remodeling processes, including neurogenesis, angiogenesis, axonal regeneration, and neural circuit reorganization. Emerging evidence demonstrates that beyond mitigating acute oxidative and inflammatory lesions, EGCG modulates endogenous repair programs to facilitate cerebral remodeling and improve persistent functional outcomes.

Promotion of endogenous neurogenesis constitutes a core reparative mechanism of EGCG. In adult mouse ischemic stroke models, delayed EGCG delivery during the recovery phase stimulates proliferation of subventricular zone (SVZ) neural progenitor cells, accelerates neuroblast migration, enhances newborn neuron survival, and improves neurobehavioral performance. These findings indicate that EGCG ameliorates the post-stroke microenvironment and boosts intrinsic cerebral neurogenesis, thereby providing novel cellular substrates for functional restoration (Zhang et al., 2017).

Effective neural repair depends on synchronized revascularization; thus, the stimulation of angiogenesis and the reconstitution of neurovascular coupling are indispensable components of EGCG’s reparative repertoire. Bai et al. demonstrated that EGCG activates Nrf2 signaling to amplify VEGF/VEGFR2-mediated angiogenic responses, increasing the number of CD31/Ki67 double-positive nascent microvessels, elevating regional microvascular density, and improving cerebral perfusion to provide sustained trophic support for newborn neurons and tissue repair (Bai et al., 2017).

In addition to neurogenesis and angiogenesis, EGCG participates in the remodeling of neural circuits. Non-stroke preclinical studies suggest EGCG facilitates neurite outgrowth and sustains neuronal structural integrity *via* the Integrin β1/FAK/p38 cascade. Yet, direct validation of this pathway’s involvement in post-stroke axonal regeneration remains absent (Zhang Y. et al., 2021). Meanwhile, EGCG modulates neurotrophic signaling and regulates synaptic protein expression, potentially promoting synaptic plasticity and reconstruction of functional neural circuits. However, evidence supporting EGCG-mediated synaptic remodeling is largely derived from non-stroke models or indirect assays, and the precise mechanisms underlying EGCG-mediated synaptic remodeling during stroke recovery require further clarification (Mandel et al., 2008).

Dynamic shifts in the immune microenvironment continuously shape cerebral remodeling. Recent studies report that EGCG modulates microglial polarization, suppresses pro-inflammatory signaling, and attenuates aberrant phagocytic activity, thereby establishing a repair-permissive immune milieu. Relevant data are nonetheless limited to *in vitro* and non-stroke platforms; the exact mechanisms by which EGCG regulates microglia to facilitate post-stroke neural repair await further characterization (Regan et al., 2024).

As research progresses, EGCG’s functional spectrum has expanded from acute neuroprotection to chronic cerebral remodeling. Stimulation of neurogenesis, enhancement of neurovascular coupling, and promotion of synaptic plasticity collectively underpin its capacity to improve long-term neurological outcomes. Beyond central nervous system protective and reparative actions, EGCG modulates peripheral platelet activation and thrombosis, offering an alternative perspective to evaluate its comprehensive interventional value in ischemic stroke onset and recurrence.

### 2.7 EGCG inhibits platelet activation and thrombosis

The onset and recurrence of ischemic stroke are tightly correlated with excessive platelet activation and thrombus formation. Antiplatelet therapy forms the cornerstone of primary and secondary ischemic stroke prevention; however, long-term administration of aspirin and P2Y12 antagonists carries inherent bleeding risks and interindividual drug resistance. Natural phytochemicals that combine antithrombotic and neuroprotective properties have therefore attracted considerable research interest (Kleindorfer et al., 2021; Greco et al., 2023).

Current evidence supporting EGCG’s antiplatelet activity falls into three tiers: *in vitro* isolated platelet assays, *ex vivo* human whole-blood studies, and *in vivo* preclinical animal experiments. *In vitro* studies utilize purified human/rodent platelets to delineate direct EGCG-mediated regulation of intracellular platelet signaling; *ex vivo* whole-blood assays preserve native cellular and humoral interactions to evaluate EGCG’s modulatory effects on platelet function; *in vivo* animal models further validate systemic antithrombotic efficacy and concurrent neuroprotection. Preclinical animal data confirm that EGCG suppresses platelet aggregation by targeting PLCγ2/Ca^2+^, cAMP/PKA/VASP, and p38 MAPK cascades to attenuate thrombus formation (Jin et al., 2008; Ok et al., 2012; Iida et al., 2014).

In *ex vivo* human whole-blood multiple-electrode aggregometry assays, Joo et al. demonstrated that EGCG robustly inhibits ADP- and collagen-induced platelet aggregation and potentiates the antiplatelet effects of aspirin, clopidogrel, and ticagrelor. These observations reflect only modulation of isolated *ex vivo* platelet function and cannot be directly extrapolated to clinical efficacy in stroke patients (Joo et al., 2018).

In summary, EGCG exhibits potential antiplatelet and antithrombotic effects by modulating multiple signaling pathways involved in platelet activation. Nevertheless, relevant evidence to date is largely limited to *in vitro* tests, *ex vivo* assays, and preclinical animal studies, and high-quality randomized controlled trials using purified EGCG remain scarce (Greco et al., 2023). Notably, existing evidence regarding the antiplatelet effects of EGCG is predominantly derived from studies on ischemic stroke and other thrombotic disorders. Sufficient data concerning its safety and applicability in hemorrhagic stroke are still lacking, and the correlation between neuroprotective dosages and antiplatelet dosages remains undefined.

## 3 Advances in EGCG nanomedicine delivery systems

### 3.1 Liposomes

Liposomes are bilayer phospholipid vesicles characterized by superior biocompatibility, dual hydrophilic/lipophilic cargo encapsulation capacity, and facile surface functionalization. EGCG is susceptible to oxidative degradation triggered by pH fluctuations, temperature, oxygen, and metal ions, while exhibiting minimal oral bioavailability and limited cerebral penetration (Lin et al., 2007; Krupkova et al., 2016). Liposomal encapsulation improves EGCG chemical stability, enables tunable sustained release, and enhances cellular uptake, thereby comprehensively optimizing its pharmaceutical performance (de Pace et al., 2013; Chen et al., 2019; Kim et al., 2024).

Multiple investigations have validated the delivery advantages of EGCG-loaded liposomes. Kim et al. fabricated gelatin-core liposomes with elevated EGCG encapsulation efficiency, superior storage stability, and sustained-release profiles, thereby boosting cellular permeability and antioxidant activity (Kim et al., 2024). Chen et al. co-encapsulated EGCG and quercetin within liposomes to achieve synergistically augmented antioxidant potency (Chen et al., 2019). In preclinical models of breast cancer, diabetes, and myocardial infarction, liposomal EGCG exhibits higher cellular uptake efficiency and more potent antioxidant and tissue-protective effects than free EGCG (de Pace et al., 2013; Bulboaca et al., 2020; Lei et al., 2023). These studies substantiate the use of liposomes as viable platforms to overcome EGCG’s stability and bioavailability limitations.

In stroke research, liposomes exploit transient ischemia-induced BBB hyperpermeability to augment cerebral drug accumulation. Al-Ahmady et al. identified two temporally distinct liposomal enrichment windows (early and delayed) post-cerebral ischemia, demonstrating that dynamic shifts in BBB permeability create time-dependent therapeutic opportunities for nanodelivery (Al-Ahmady et al., 2019). Yang et al. further developed IGF1R-targeted salvianolic acid liposomes, which increased cerebral drug deposition, reduced infarct volume, and ameliorated neurological and inflammatory phenotypes in ischemic stroke models, thereby validating the feasibility of targeted liposomal delivery for polyphenolic neuroprotective agents (Yang et al., 2024).

In summary, EGCG liposomes display well-documented advantages in stability, sustained release, and cellular uptake. At the same time, stroke-specific research confirms liposomes augment cerebral drug delivery *via* passive BBB opening or active receptor targeting. Future work should systematically compare the intracerebral pharmacokinetics and safety profiles of free EGCG, conventional liposomes, PEGylated liposomes, and brain-targeted liposomes, and optimize administration timing based on dynamic alterations in post-stroke BBB permeability.

### 3.2 Polymeric nanoparticles

Polymeric nanoparticles are predominantly synthesized from biodegradable matrices, including poly(lactic-co-glycolic acid) (PLGA), PEG-modified PLGA (PEG-PLGA), and chitosan, which feature excellent biocompatibility, high drug-loading capacity, and tunable release kinetics. For EGCG, which readily undergoes oxidative degradation, polymeric nanocarriers improve encapsulation yield, prolong systemic circulation half-life, redistribute tissue biodistribution, and enhance cellular internalization, thereby elevating overall therapeutic efficacy (Sanna et al., 2011; Tyagi et al., 2017; Bhattacharya et al., 2024).

EGCG-loaded polymeric nanoparticles exhibit promising translational potential in stroke models. Yang et al. adopted a double-emulsion technique to synthesize PEG-PLGA nanoparticles encapsulating EGCG (EGCG-NPs) with an average particle size of 167 nm and encapsulation efficiency of 86%, enabling sustained cargo release over approximately 8 days. Relative to free EGCG, EGCG-NPs demonstrate stronger antioxidant activity and negligible cytotoxicity. Co-administration of EGCG-NPs with nimodipine (first-line standard SAH therapy) elicits robust synergistic neuroprotection in experimental SAH models: combined treatment alleviates Ca^2+^ overload, suppresses oxidative stress and mitochondrial dysfunction, restores autophagic equilibrium, and reduces neuronal apoptosis, yielding superior improvements in cerebral pathological lesions and neurological function compared with monotherapy using either agent alone (Yang et al., 2023). This landmark study provides direct preclinical evidence that PEG-PLGA nanodelivery elevates EGCG bioavailability and potentiates synergistic neuroprotection with nimodipine for SAH treatment.

Beyond stroke platforms, polymeric nanoparticle-mediated EGCG delivery enhances chemical stability, extends release duration, improves cellular uptake, and boosts brain targeting across alternative disease models. These investigations primarily provide insights into formulation and delivery rather than stroke-specific therapeutic data, necessitating further validation of efficacy and safety in the context of cerebrovascular disease (Lv et al., 2020; Cano et al., 2021).

### 3.3 Exosomes

Exosomes are 30–150 nm extracellular vesicles endowed with exceptional biocompatibility, low immunogenicity, and intrinsic capacity to traverse the BBB, ranking among the most translationally promising natural nanocarriers (Kalluri and LeBleu, 2020; O’Brien et al., 2020). Unlike synthetic nanoparticles, exosomes retain membrane proteins and bioactive cargo from their parent cells, facilitating intercellular communication while improving drug stability, tissue tropism, and cerebral delivery efficiency (Xin et al., 2013).

A wealth of stroke preclinical studies confirms that exosomes themselves exert potent neuroprotective effects. Mesenchymal stem cell (MSC), neural stem cell, and endothelial progenitor cell-derived exosomes deliver microRNAs, proteins, and lipids to mitigate neuroinflammation, suppress neuronal apoptosis, promote angiogenesis, and facilitate neural repair to improve post-ischemic functional recovery (Doeppner et al., 2015). Recent studies have also shown that MSC-derived exosomes possess intrinsic anti-inflammatory and tissue repair properties in regenerative medicine, further supporting their potential as therapeutic nanocarriers, although direct evidence in stroke remains limited. These findings provide additional indirect support for the development of exosome-based EGCG delivery systems while emphasizing that validation in stroke-specific models is still required (Abd-Rabou et al., 2025).

Current research combining EGCG and exosomes predominantly focuses on cardiovascular and tissue regeneration fields. Zhang et al. reported that EGCG pretreatment of ischemic cardiomyocytes stimulates the secretion of miR-30a-enriched exosomes, which inhibit cardiomyocyte apoptosis and pathological autophagy, thereby ameliorating cardiac dysfunction following acute myocardial infarction, indicating that EGCG modulates exosome-mediated intercellular signaling alongside its direct antioxidant effects (Zhang C. et al., 2020).

Advancements in exosome engineering have enabled the loading of EGCG into extracellular vesicles. A recent investigation used menstrual blood-derived exosomes to encapsulate EGCG, achieving an encapsulation efficiency of ∼65%; exosomal EGCG significantly enhanced cellular adhesion capacity and upregulated endometrial receptivity-related genes compared with free EGCG, thereby demonstrating that exosomes improve EGCG stability and tissue targeting (Rasool et al., 2025).

Collectively, stroke research robustly establishes exosomes as superior brain-targeted carriers. At the same time, EGCG-focused studies demonstrate EGCG can regulate exosomal biological function and be encapsulated as therapeutic cargo to augment tissue accumulation. Engineering brain-derived, or EGCG-loaded modified exosomes represents a promising precision delivery strategy for stroke. Future research must systematically characterize the distribution of cerebral tissue, pharmacokinetic profiles, and long-term neuroprotective safety of exosomal EGCG formulations.

### 3.4 Metal–organ frameworks (MOFs)

Metal–organic frameworks (MOFs) are porous, crystalline materials self-assembled *via* coordination bonds between metal ions/clusters and organic ligands, featuring ultrahigh specific surface areas, tunable pore dimensions, high drug-loading capacity, and facile surface functionalization, and have emerged as cutting-edge multifunctional nanoplatforms in recent years (Hefayathullah et al., 2024). MOFs enhance the encapsulation efficiency and chemical stability of natural bioactive compounds *via* host-guest interactions and surface modification, enabling stimuli-responsive cargo release and tissue-targeted delivery, with broad applications in drug delivery, molecular imaging, and combinatorial therapy (Hefayathullah et al., 2024; Wang D. et al., 2024).

Substantial progress has been achieved in MOF-based nanotherapeutics for stroke intervention. Min et al. fabricated fucoidan-coated UiO-67 MOF nanoparticles (Fu@UiO-67) for localized nimodipine delivery post SAH (Min et al., 2026). Fucoidan confers thrombus-targeting properties, prolonging nanoparticle retention within the subarachnoid space and elevating cerebrospinal fluid drug exposure to enable sustained release. Compared with free nimodipine, Fu@UiO-67 markedly alleviates cerebral vasospasm, improves cerebral perfusion and neurological function, and reduces systemic hypotensive adverse effects, highlighting the advantages of MOFs for localized, precise delivery in SAH.

Beyond canonical MOFs, metal-phenolic networks (MPNs), a subclass of MOF-derived nanomaterials, have been increasingly deployed for stroke therapy. Liu et al. self-assembled EGCG and Fe^3+^ to construct metal-phenolic nanoparticles, further cloaked with macrophage membranes to generate membrane-camouflaged M@EFE nanomedicines (Liu et al., 2026). This nanoplatform integrates inflammatory tropism and cerebral enrichment capacity, efficiently crosses the BBB, synergistically scavenges ROS, suppresses lipid peroxidation and neuroinflammation, and reduces neuronal apoptosis. In mouse MCAO ischemic stroke models, M@EFE NPs drastically shrink infarct volume, accelerate neurological recovery, and prolong long-term survival, demonstrating robust therapeutic potential for ischemic stroke.

Overall, MOFs and MPN-based nanomedicines exhibit favorable attributes, including brain targeting, sustained release, and multimodal combinatorial therapy; nevertheless, research on EGCG-loaded MOFs for stroke remains in the early preclinical stage. Future directions include developing ROS/pH-responsive and brain-ligand-functionalized EGCG-MOF constructs, alongside systematic evaluation of cerebral biodistribution, pharmacokinetics, and long-term biosafety to accelerate translational progress. A comparison of representative EGCG-based nanodelivery platforms for stroke therapy is summarized in Table 1.

**TABLE 1:** Comparison of representative EGCG-Based nanodelivery platforms for stroke therapy.

Platform	Representative formulations	Stroke-related evidence	BBB penetration/targeting	Major delivery and pharmacological effects	Advantages	Limitations	Evidence level/translational readiness	​
Liposomes	Conventional, PEGylated, gelatin-core, receptor-targeted	Indirect preclinical evidence	Passive BBB opening; IGF1R-mediated active targeting	Improved stability, prolonged circulation, sustained release, enhanced uptake	Mature, biocompatible, easy surface modification	Leakage, oxidation, limited PK data	Indirect preclinical; high maturity	Al-Ahmady et al. (2019), Yang et al. (2024), Sanna et al. (2011), Tyagi et al. (2017), Bhattacharya et al. (2024), Yang et al. (2023)
Polymeric NPs	PLGA, PEG–PLGA, chitosan	Direct preclinical evidence	PEG prolongation; passive accumulation; ligand targeting	Controlled release; anti-oxidative stress; autophagy restoration	High loading; tunable degradation	Need safety/PK validation	Strongest preclinical evidence	Cano et al. (2021), Kalluri and LeBleu (2020), O’Brien et al. (2020)
Exosomes	MSC-, NSC-, EPC-derived; engineered	Carrier evidence strong; EGCG indirect	Intrinsic BBB crossing; engineered homing	Improved bioavailability; anti-inflammatory; pro-angiogenic	Excellent biocompatibility	Low yield; heterogeneity	High translational promise	Wang D. et al. (2024), Min et al. (2026), Liu et al. (2026)
MOFs/MPNs	UiO MOFs, fucoidan-coated MOFs, EGCG–Fe3+ MPNs	Emerging direct preclinical evidence	Fucoidan targeting; ROS/pH-responsive	ROS scavenging; anti-ferroptosis; sustained delivery	High loading; stimuli-responsive	Complex synthesis; metal safety	Early translational stage	Feigin et al., (2023), Wang et al. (2023), Yang and Wang (2016), O’Donnell et al. (2016)

## 4 Clinical translational prospects and current evidence of EGCG

### 4.1 EGCG for long-term chronic stroke risk management

Contemporary stroke prevention and management paradigms have evolved from exclusive acute-phase intervention to full-cycle chronic disease control encompassing risk factor modification, lifestyle adjustment, and long-term health surveillance. Cumulative evidence confirms that modifiable risk factors including hypertension, diabetes, dyslipidemia, obesity, and chronic low-grade inflammation synergistically drive stroke incidence and recurrence, making comprehensive vascular risk reduction the cornerstone of primary and secondary stroke prevention (Feigin et al., 2023; Greco et al., 2023; GBD 2021 Stroke Risk Factor Collaborators, 2024). Given EGCG’s antioxidant, anti-inflammatory, endothelium-protective, and glucose- and lipid-regulating bioactivities, it holds promise as an adjunct intervention for integrated cerebrovascular risk management to reduce the risk of stroke onset and recurrence.

Multiple prospective cohort meta-analyses link green tea consumption to reduced stroke risk. A meta-analysis incorporating 16 prospective cohorts enrolling 645,393 participants demonstrated that green tea intake correlates with a 26% relative reduction in stroke risk (RR = 0.74, 95% CI 0.68–0.80), with significant non-linear dose-response associations (P < 0.001): daily consumption of ∼300 mL green tea corresponds to a 16% relative risk reduction compared to non-consumers (Wang et al., 2023). However, these observational studies only establish correlation rather than causal inference. Green tea contains a complex mixture of EGCG, EGC, ECG, EC, theanine, and caffeine; the cerebroprotective benefits of whole green tea cannot be singularly attributed to EGCG, nor can equivalent therapeutic efficacy be assumed for purified EGCG supplementation (Yang and Wang, 2016).

While definitive human interventional data remain limited, preclinical investigations support EGCG’s capacity to ameliorate stroke-associated vascular pathology. Hypertension constitutes the primary modifiable stroke risk factor driving atherosclerosis, cerebral small vessel disease, and BBB disruption (O’Donnell et al., 2016; Greco et al., 2023). In angiotensin II-infused hypertensive murine models, EGCG significantly reduces systolic blood pressure, alleviates oxidative stress, and attenuates eNOS uncoupling to restore nitric oxide (NO) bioavailability and improve endothelial function (Mohd Sabri et al., 2022). Meta-analyses of randomized controlled trials indicate that sustained green tea intake for 8 weeks or more modestly lowers systolic and diastolic blood pressure. However, these outcomes reflect whole-tea interventions rather than purified EGCG monotherapy (Peng et al., 2014).

Dyslipidemia represents another major stroke risk factor accelerating atherosclerotic plaque formation and cerebral arterial stenosis. While statins remain first-line lipid-lowering agents, EGCG, a natural phytochemical, exhibits robust antioxidant and anti-inflammatory properties. Research confirms that EGCG inhibits oxidative modification of low-density lipoprotein cholesterol (LDL-C), suppresses foam cell formation, and retards atherosclerotic progression (Kim et al., 2014; Onakpoya et al., 2014). Combined with EGCG’s documented endothelial protection, anti-inflammatory, antiplatelet, and NVU-stabilizing effects, it theoretically mitigates multi-factorial vascular injury and thrombotic risk as an adjunct for secondary stroke prevention. Nevertheless, EGCG cannot replace statins, antihypertensives, or antiplatelet agents recommended by clinical guidelines and should only be used as a a supplementary intervention (Greenberg et al., 2022; Greco et al., 2023).

Advancements in nanomedicine deliver novel translational avenues for EGCG. Liposomal, polymeric, and MOF-based EGCG nanoformulations enhance chemical stability and cerebral delivery efficiency. Preclinical SA studies reveal that EGCG nanoparticles mitigate ferroptosis, oxidative stress, and neuronal loss; in ischemic stroke models, novel nanocarriers augment cerebral targeting and neuroprotective potency (Yang et al., 2023; Huang et al., 2024; Liu et al., 2026). Despite these promising preclinical observations, clinical data evaluating EGCG nanomedicines for stroke recurrence, post-stroke cognitive dysfunction, and long-term functional recovery are currently lacking.

In summary, evidence supporting EGCG for chronic stroke risk management is predominantly derived from basic research, while epidemiological data only reflect whole green tea consumption rather than purified EGCG supplementation. Future large-scale randomized controlled trials enrolling high-risk stroke populations and stroke survivors are required to evaluate EGCG’s long-term impacts on stroke recurrence, neurological function, cognitive status, and quality of life, alongside standardized optimal dosing, timing, and combinatorial safety profiles. With further refinement of high-bioavailability nanocarriers, EGCG nanoformulations may emerge as valuable adjuncts for full-cycle stroke management.

### 4.2 EGCG for acute stroke therapy

Stroke, particularly ischemic stroke, exhibits an intricate, multilayered pathophysiology involving diverse cellular and molecular cascades. During the acute phase, infarcted tissue releases abundant matrix metalloproteinases (MMPs) and pro-inflammatory cytokines, thereby activating microglia and initiating reciprocal inflammatory and repair signaling (Wang C. et al., 2024). Cumulative preclinical data confirm EGCG exerts robust acute neuroprotection *via* activating the Keap1/P62/Nrf2 axis to suppress oxidative stress, iron deposition, and neuronal apoptosis. In rat ICH models, administration of 10 mg/kg EGCG reduces hematoma volume by approximately 30% (Hao et al., 2024). Damage-associated molecular patterns (DAMPs) liberated from ischemic lesions activate immune cells to secrete pro-inflammatory mediators and exacerbate cerebral injury; EGCG blunts NF-κB signaling to reduce cytokine secretion and alleviate inflammatory brain damage (Kim et al., 2022). EGCG-NPs suppress excessive HO-1 activation to remodel iron metabolism and inhibit ferroptosis (Huang et al., 2024). In SAH models, HO-1 mRNA expression is markedly upregulated compared with sham groups (p < 0.01), whereas EGCG-NP pretreatment significantly represses HO-1 transcription. Furthermore, EGCG-NPs modulate the iron regulatory proteins TFR1, DMT1, and FPN to restore iron homeostasis, while upregulating GPX4, GSH, and FSP1 to inhibit ferroptosis and ferritin degradation, thereby identifying novel intervention targets for SAH management. EGCG restores NKA activity and elevates GLT-1 expression to rectify ionic imbalance and mitigate excitotoxicity across multiple stroke animal models, reducing infarct volume and improving neurological function. However, divergent experimental models, treatment timing, and dosage ranges across studies preclude the establishment of a universal optimal therapeutic dose; EGCG’s neuroprotective potency is dose-dependent and modulated by experimental conditions (Liu et al., 2025). Transient MCAO and OGD/R induce pathological autophagy; EGCG administration reduces infarct volume and neuronal loss *in vivo* in MCAO mice, while attenuating OGD/R-mediated cell death *in vitro* in HT22 neurons *via* inhibiting autophagy through AKT/AMPK/mTOR phosphorylation signaling (Wang et al., 2022a).

Despite rt-PA and mechanical thrombectomy constituting first-line acute ischemic stroke therapies, both modalities carry substantial limitations (Wang H. et al., 2024). Rt-PA has a short systemic half-life and is restricted to patients presenting within 4.5 h of symptom onset; eligible candidates for mechanical thrombectomy may receive intervention up to 24 h after symptom onset (Mizuma and Yenari, 2021). Rt-PA administration beyond the 4.5-h window is contraindicated due to elevated risks of intracranial hemorrhage and excitotoxic neuronal injury (Xu et al., 2021). Research indicates that combined EGCG and rt-PA therapy reduces MMP-2 and MMP-9 levels, potentially prolonging rt-PA’s effective onset-to-treatment window, improving NIHSS scores, and enhancing patient functional recovery (Pacifici et al., 2021). Nevertheless, this finding originates from exploratory, small-sample clinical research and subgroup analyses. It lacks definitive confirmation that EGCG safely expands the rt-PA therapeutic window or improves long-term functional endpoints. Corresponding ischemic stroke animal studies further validate that that EGCG mitigates delayed rt-PA-induced BBB disruption, MMP activation, and hemorrhagic transformation, providing mechanistic support for adjunct EGCG therapy (You, 2016). Collectively, current evidence suggests that EGCG is a promising rt-PA adjuvant, yet large multicenter randomized controlled trials are required for definitive validation.

## 5 Challenges and solutions

### 5.1 Core challenges in the clinical translation of EGCG

#### 5.1.1 Limited bioavailability

Low bioavailability remains one of the major obstacles to the clinical translation of EGCG. From the perspective of absorption, the oral bioavailability of free EGCG is extremely low, with peak plasma concentrations reaching only approximately 0.15 μM following oral administration (Wang Y. et al., 2022; Kong et al., 2024). This indicates that only a small fraction of orally administered EGCG enters the systemic circulation, whereas the majority is not effectively absorbed. In addition, EGCG exhibits poor chemical stability under physiological conditions, particularly in the gastrointestinal tract. In simulated intestinal fluid (pH 6.8), the retention rate of free EGCG is only 14.1%, indicating that it is highly susceptible to degradation and has difficulty maintaining its structural integrity and biological activity (Kothari et al., 2024). Furthermore, EGCG has a relatively short elimination half-life of less than 4 h, which further limits its duration of action *in vivo* (Zhang S. et al., 2020).

These unfavorable pharmacokinetic characteristics may prevent EGCG from achieving therapeutically effective concentrations in brain tissue, thereby limiting its sustained regulation of oxidative stress, neuroinflammation, and blood–brain barrier (BBB) disruption after stroke. Therefore, improving brain delivery efficiency remains a critical challenge for the clinical translation of EGCG.

#### 5.1.2 Blood–brain barrier constraints

The blood–brain barrier (BBB) is a highly selective biological barrier composed of brain microvascular endothelial cells connected by tight junctions, together with the basement membrane, pericytes, and astrocytes (Minnelli et al., 2020). Its primary physiological function is to protect the central nervous system from harmful substances while maintaining cerebral homeostasis. Under normal conditions, the BBB effectively prevents bacteria, viruses, and most therapeutic agents from entering the brain parenchyma, thereby preserving normal neurological function. However, this highly selective barrier also limits the delivery of EGCG to the brain.

Importantly, the BBB does not completely prevent EGCG from entering brain tissue, but the resulting cerebral exposure remains relatively low. Following oral administration of a high dose of EGCG in rats, approximately 0.5 nmol/g EGCG was detected in brain tissue, corresponding to an estimated total tissue concentration of approximately 0.5 μM when assuming a brain tissue density of 1 g/mL. Repeated administration increased the EGCG-related signal in the brain by approximately sixfold, which would theoretically correspond to about 3 μM. However, this signal may also include EGCG metabolites, protein-bound forms, and degradation products. It therefore should not be interpreted as the concentration of pharmacologically active free EGCG within the brain interstitial space (Youn et al., 2022).

Furthermore, an *in vitro* BBB model demonstrated that the permeability of EGCG was only approximately 2.8% within 30 min, indicating that although EGCG can cross the BBB, its transport efficiency remains limited (Pervin et al., 2017). These findings indicate that EGCG can reach brain tissue, but its cerebral exposure remains limited. Consequently, improving BBB penetration, enhancing lesion-targeted accumulation, and prolonging local retention have become major objectives in the development of EGCG-based nano-delivery systems.

#### 5.1.3 Safety concerns

The safety profile of EGCG deserves careful consideration, particularly under certain physiological or clinical conditions. Current evidence indicates that the safety of EGCG is strongly dose-dependent. Human intervention studies have shown that purified EGCG is generally well tolerated at moderate doses, whereas long-term administration of high doses may increase the risk of liver injury. Therefore, future clinical studies in stroke should further define the optimal dosage, treatment duration, and long-term safety profile of EGCG (Yang et al., 2020).

Clinical studies have reported that oral administration of purified EGCG or standardized green tea extracts at doses of approximately 200–400 mg/day is generally well tolerated. However, the European Food Safety Authority (EFSA) has concluded that intake of concentrated EGCG supplements at doses≥800 mg/day is associated with an increased risk of elevated serum transaminase levels. Nevertheless, this risk is influenced by formulation type, fasting status, treatment duration, and individual susceptibility and therefore should not be regarded as a universal toxicity threshold (EFSA Panel on Food Additives and Nutrient Sources added to Food (ANS) et al., 2018; Hu et al., 2018).

In contrast, acute toxicity studies in mice employed substantially higher doses, including a single oral dose of 1,500 mg/kg or 750 mg/kg/day for two consecutive days, resulting in marked hepatocellular necrosis and elevated alanine aminotransferase (ALT) levels. These findings reflect acute high-dose exposure rather than clinically relevant therapeutic dosing in humans (Lambert et al., 2010). Overall, EGCG appears to exhibit a favorable safety profile at clinically relevant doses, although long-term administration should still be accompanied by regular monitoring of liver function.

### 5.2 Innovative strategies to break through translational barriers

#### 5.2.1 Formulation optimization: the rise of nanotechnology

Nanotechnology has opened up transformative avenues to resolve the translational obstacles of EGCG, among which nano-formulated EGCG represents a landmark breakthrough. Singh et al. pioneered PLA-PEG-based EGCG nanoparticles (nanoEGCG), which effectively address the inherent defects of free EGCG, including poor chemical stability and low systemic bioavailability. In in vivo assays, this nanoformulation increased plasma drug concentration by 1.4-fold and cerebral concentration by 1.6-fold (Kong et al., 2024). In addition to PLA-PEG polymeric nanoparticles, researchers have explored a wide range of nanocarriers to enhance the pharmacological potency of EGCG. Polymeric nanoparticles stand out as a popular candidate owing to favorable biocompatibility and biodegradability. They encapsulate EGCG to shield it from external degradation, thus enhancing molecular stability. Moreover, surface modification of polymeric nanoparticles enables targeted delivery, transporting EGCG precisely to lesion sites to improve therapeutic outcomes.

Micelles are another widely adopted nanocarrier type, self-assembled from amphipathic molecules into nanoscale aggregates under aqueous conditions. EGCG can be encapsulated within the hydrophobic core of micelles to strengthen its aqueous solubility and chemical stability. Meanwhile, the small particle size and uniform dispersibility of micelles facilitate transmembrane penetration, thereby accelerating EGCG absorption and delivery.

In addition, researchers synthesized quaternized chitosan (HTCC) and conjugated it with fucoidan (FD) or low-molecular-weight fractions (LMWF) to fabricate EGCG-loaded nanoparticles. Such nanoparticles exhibit outstanding stability in simulated gastric and intestinal fluids, with encapsulation efficiencies of 46.1% and 42.4%, respectively. Their permeability across Caco-2 monolayers is 1.9–2.1 times higher than that of free EGCG (Kothari et al., 2024). When EGCG is loaded into nanospanlastics, rat *in vivo* experiments demonstrate superior peak plasma concentration (Cmax = 0.58 μg/mL), larger area under the curve (AUC_0-24_ = 6.54 μg h/mL), and prolonged half-life (t_1_/_2_ = 17.43 h) compared with conventional niosomes and free EGCG suspensions (Man et al., 2020). Green synthetic approaches are employed to prepare gold nanoparticle-decorated EGCG nanospheres (EGCG NS@AuNPs), which exhibit excellent stability in high-salt media and low cytotoxicity against L929 cells (Kuo et al., 2021). Collectively, all types of nanodelivery platforms improve EGCG stability, bioavailability and tissue-targeting capacity, offering novel tactics to enhance cerebral drug exposure and facilitate clinical translation. Nevertheless, most relevant data originate from pharmaceutical formulation studies or non-stroke disease models; their long-term safety, cerebral pharmacokinetic profiles, and clinical efficacy in stroke treatment remain to be systematically validated (Wu et al., 2022).

#### 5.2.2 Chemical structural modification

Extensive chemical modification attempts have been conducted to optimize the physicochemical properties of EGCG, among which lipophilic derivatization is a vital research direction. C18-EGCG is a representative of this strategy. Chemical lipophilic modification markedly strengthens the membrane affinity of EGCG and confers superior protective effects under oxidative stress. Although its free-radical scavenging capacity is only around 65% (vs. 95% for native EGCG), it increases cellular viability by approximately 41% under oxidative stress (Wu et al., 2022). This phenomenon indicates that lipophilic derivatization slightly compromises radical-scavenging activity but enhances cytoprotective effects by increasing membrane-binding affinity, laying new foundations for EGCG applications. In addition to C18-EGCG, a series of 4″-alkyl ether derivatives (4″-Cn EGCG, where n is an even integer ranging from six to 18) have been synthesized. All derivatives exert stronger antioxidant activity than vitamin C, with IC_5_0 values ranging from 6.16 to 15.76 µM, while the IC_5_0 of vitamin C is 18.50 µM (Mazyed et al., 2021). These results fully verify that structural chemical modification can effectively augment the antioxidant capacity of EGCG analogues and broaden their application prospects in antioxidant therapy. The design of fluorinated zinc-coordinated nanoassemblies represents another innovative advance in EGCG chemical engineering. Such nanoassemblies drastically enhance membrane affinity and delivery efficiency, achieving a drug-loading rate of approximately 30% when co-loaded with chemotherapeutic agents such as sorafenib (Wu et al., 2025). This characteristic enables EGCG to exert intrinsic bioactivities while acting as a co-delivery carrier for synergistic combination therapy.

Furthermore, ProEGCG synthesized *via* acetylation exhibits superior stability and bioavailability, maintaining a plasma concentration of 1.36 μg/mL for up to 360 min in mice (Cleeland et al., 2012). Such modified derivatives and supramolecular assemblies offer multiple opportunities for the clinical translation of EGCG by addressing the drawbacks of poor bioavailability and low delivery efficiency, thereby enabling EGCG to act on lesion sites more effectively. Their favorable drug-loading capacity also facilitates combining medications to generate synergistic therapeutic effects. Continuous innovations in EGCG structural modification provide robust technical support for its bench-to-bedside transformation.

#### 5.2.3 Combination therapeutic regimens

The pathological cascade of stroke involves mutually coupled events including oxidative stress, neuroinflammation, blood–brain barrier disruption and mitochondrial dysfunction, as well as cell death pathways. Monotherapy often fails to achieve sufficient neuroprotective outcomes. Accordingly, combining EGCG with standard stroke therapies represents a critical translational direction, which may simultaneously amplify reperfusion benefits and alleviate secondary brain injury.

In acute ischemic stroke, existing clinical evidence mainly focuses on the combined administration of EGCG and recombinant tissue plasminogen activator (rt-PA). A randomized, double-masked, placebo-controlled trial reported that adjuvant EGCG therapy was associated with improved NIHSS scores and reduced plasma MMP-2/MMP-9 levels in delayed-treatment subgroups (Wang and You, 2017). However, this trial suffers from limited sample size and relies on subgroup and surrogate markers for efficacy evaluation. At present, it cannot be confirmed whether EGCG can safely extend the rt-PA therapeutic window or improve long-term functional endpoints. EGCG can only be regarded as a candidate adjunct neuroprotector rather than a replacement for guideline-recommended intravenous thrombolysis (Wang and You, 2017; Powers et al., 2019). The trial itself positions EGCG as supplementary therapy for rt-PA, yet its conclusions require verification *via* larger-scale clinical trials.

In subarachnoid hemorrhage (SAH), direct preclinical evidence supports the combined use of EGCG nanoparticles and nimodipine. Yang et al. prepared PEGylated PLGA EGCG nanoparticles and co-administered them with nimodipine in experimental SAH models. Compared with monotherapy of EGCG nanoparticles or nimodipine alone, combinatorial intervention further relieves Ca^2+^ overload, oxidative stress and mitochondrial dysfunction, suppresses aberrant autophagy and reduces neuronal loss to improve neurological function, which suggests that elevated EGCG bioavailability amplifies synergistic neuroprotection with nimodipine (Yang et al., 2023). Nevertheless, this study uses pre-treatment administration, and all data are derived from animal experiments, which cannot be directly extrapolated to patients with acute SAH. Nimodipine remains the standard guideline-approved drug for aneurysmal SAH, while EGCG combination therapy is still in the preclinical exploratory stage (Hoh et al., 2023).

In summary, current evidence supporting combined EGCG treatment for stroke is limited to preliminary clinical trials of rt-PA adjuvant therapy and animal studies of EGCG nanoparticles combined with nimodipine for SAH. Future research should prioritize standard therapies with definite stroke indications and systematically evaluate the safety, optimal timing of treatment, and incremental benefits of EGCG combined with intravenous thrombolysis, endovascular thrombectomy, or nimodipine. Clinical endpoints such as 90-day modified Rankin Scale, all-cause mortality, recurrent hemorrhage, symptomatic intracranial hemorrhage and delayed cerebral ischemia should be adopted instead of referencing combinatorial efficacy data from oncology, Parkinson’s disease or general cognitive impairment research.

## 6 Discussion and future perspectives

Stroke is a heterogeneous disorder driven by reciprocal crosstalk among neurons, glial cells, cerebral microvascular endothelial cells, pericytes, infiltrating immune cells, and the extracellular matrix, with pathological manifestations that reflect global neurovascular unit (NVU) dysfunction. A growing body of research confirms that single-target interventions cannot fully block post-stroke secondary brain injury; simultaneous modulation of interconnected pathological cascades, including oxidative stress, neuroinflammation, mitochondrial dysfunction, blood–brain barrier damage, ferroptosis, excitotoxicity, and neural remodeling, has become a core developmental trend in stroke neuroprotection (Li et al., 2024; Bahr-Hosseini and Saver, 2025). EGCG exerts multiple pharmacological activities, including antioxidant, anti-inflammatory, regulation of cell death, and maintenance of NVU homeostasis, and it can remodel the cerebral microenvironment after stroke. In the era of reperfusion therapy, EGCG is more likely to serve as an adjunct neuroprotector after intravenous thrombolysis or mechanical thrombectomy, forming complementary regimens with standard recanalization therapies to further improve patient prognosis (Anderson and Song, 2024; Li et al., 2024; Schmidt-Pogoda et al., 2024; Bahr-Hosseini and Saver, 2025). Despite abundant preclinical studies confirming the promising neuroprotective potential of EGCG, multiple obstacles remain in its transition from laboratory research to clinical application.

### 6.1 Current limitations and challenges

#### 6.1.1 Discrepancies between preclinical models and real-world patients

Existing preclinical stroke research on EGCG mainly adopts young, genetically homogeneous rodents, while stroke patients in clinical practice are mostly elderly individuals complicated with hypertension, diabetes, dyslipidemia, and atherosclerosis. Such disparity creates prominent translational gaps that are widely encountered in stroke neuroprotection research (Anderson and Song, 2024; Schmidt-Pogoda et al., 2024). Patient age, gender, metabolic status and chronic inflammation can modulate oxidative stress, autophagy, immune response, BBB function and drug metabolism, thereby altering the neuroprotective effects of EGCG.

Meanwhile, most animal studies deliver EGCG before or immediately after stroke onset. In clinical scenarios, patients undergo symptom recognition, pre-hospital transport, and imaging assessment before reperfusion intervention, resulting in distinct therapeutic time windows between animal and human studies. In addition, current preclinical evaluations mainly rely on infarct volume and short-term neurological scores as endpoints, with insufficient attention to long-term outcomes such as cognitive impairment, emotional disorders, white matter injury, neurovascular remodeling and quality of life. Therefore, existing preclinical findings cannot fully reflect the real clinical conditions of elderly, multimorbid patients receiving multiple concurrent medications and standard reperfusion therapy, and the relevant conclusions require further validation in clinically relevant disease models and high-quality clinical trials (Anderson and Song, 2024; Schmidt-Pogoda et al., 2024).

#### 6.1.2 Translational barriers of nanodelivery systems

Advancements in nanotechnology provide novel strategies to overcome EGCG’s limitations in stability and cerebral targeting efficiency. However, most nanocarrier research remains at the preclinical stage, and favorable efficacy observed in laboratory settings has not translated into mature clinical products. The scaling-up transition from lab-scale preparation to industrial mass production requires consistent batch-to-batch performance of key quality attributes, including particle size distribution, morphology, drug loading, and encapsulation efficiency, as well as standardized GMP-compliant protocols for preparation, sterilization, storage, and transportation (Ji et al., 2024; Lei et al., 2024).

On the other hand, numerous novel nanocarriers employ sophisticated designs, such as biomimetic membrane coatings and receptor-targeted surface modifications, to enhance cerebral accumulation. While these strategies improve lesion-targeting capacity, they raise production costs and complicate quality control and regulatory approval processes. In addition, stroke-targeted nanomedicines generally suffer from insufficient brain penetration, incomplete long-term biosafety evaluation and barriers to mass manufacturing; systematic assessments of nanocarrier tissue accumulation, metabolic clearance, immunogenicity and repeated-dose toxicity remain scarce. These factors collectively hinder the clinical translation of EGCG nanoformulations (Ji et al., 2024; Lei et al., 2024).

#### 6.1.3 Limitations of clinical evidence

Compared with abundant cellular and animal data, direct clinical evidence supporting EGCG administration for stroke treatment remains scarce. Current human research consists of epidemiological surveys on green tea intake, pharmacokinetic trials in healthy volunteers, and small exploratory clinical trials, which cannot sufficiently demonstrate that purified EGCG improves clinical outcomes in acute stroke patients (Wang and You, 2017; Wang et al., 2023). Moreover, green tea contains a complex mixture of EGCG, ECG, EGC, EC, theanine, and caffeine, so the favorable outcomes observed in green tea-related studies cannot be attributed solely to EGCG as a monomer or extrapolated to purified EGCG monotherapy (Wang et al., 2023).

Systematic investigations are still lacking on the optimal dosage, route of administration, and therapeutic window of EGCG across various stroke subtypes, as well as its efficacy and safety when combined with thrombolytics, antiplatelets, anticoagulants, and statins. As reperfusion therapy becomes standard care for acute ischemic stroke, EGCG will function as an adjunct intervention rather than a replacement for recanalization treatments. Before clinical application, well-designed, large-sample, multicenter randomized controlled trials are mandatory to clarify its benefit-risk profile and identify eligible patient populations (Anderson and Song, 2024; Schmidt-Pogoda et al., 2024).

This figure illustrates the proposed translational roadmap of epigallocatechin-3-gallate (EGCG) from current challenges to future precision therapy for stroke. The roadmap highlights six sequential steps: (1) current limitations of free EGCG, including low oral bioavailability, poor chemical stability, rapid metabolism, and limited brain exposure; (2) optimization of nanodelivery systems, including liposomes, polymeric nanoparticles, exosomes, and metal–organic frameworks (MOFs), to improve physicochemical stability, systemic bioavailability, brain accumulation, blood–brain barrier (BBB)-targeted delivery, and stimuli-responsive release; (3) enhanced therapeutic benefits, including attenuation of secondary brain injury, preservation of BBB and neurovascular unit (NVU) integrity, and improvement of neurological function; (4) future translational strategies based on combination therapy and precision nanomedicine; (5) clinical translation through pharmacokinetic evaluation, dose optimization, biomarker-guided patient stratification, safety assessment, and randomized clinical trials; and (6) future precision therapy for stroke. This roadmap summarizes the key translational considerations discussed in this review and provides a conceptual framework for the future clinical development of EGCG-based nanomedicines. The proposed translational roadmap for EGCG nanodelivery in precision stroke therapy is illustrated in Figure 2.

**FIGURE 2:**
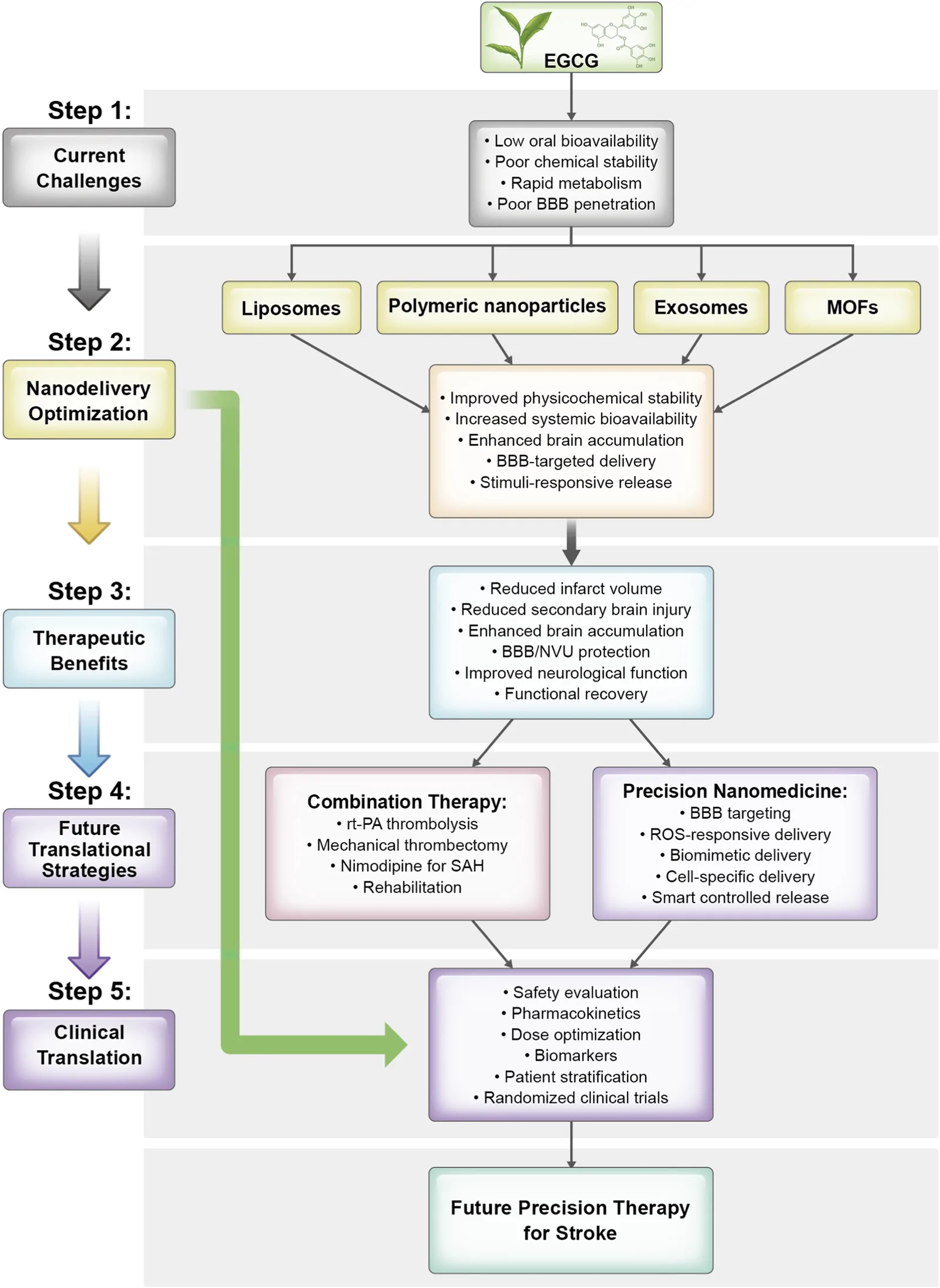
Translational roadmap of EGCG nanodelivery for precision therapy in stroke.

### 6.2 Future research recommendations

Beyond ischemic stroke, the therapeutic spectrum of EGCG can be extended to intracerebral hemorrhage (ICH), subarachnoid hemorrhage (SAH), cerebral small vessel disease (CSVD), vascular cognitive impairment (VCI) and post-stroke depression (PSD). All these cerebrovascular disorders share overlapping pathological signatures including persistent oxidative stress, chronic neuroinflammation, ferroptosis, microvascular damage and NVU disruption. Future research should explore whether EGCG modulates these shared cascades to develop unified interventions applicable to all stroke subtypes and their neurological sequelae, advancing precision cerebrovascular therapy (Hao et al., 2024; Li et al., 2024; Kamboj et al., 2025; Yang et al., 2025).

To facilitate the bench-to-bedside translation of EGCG for stroke treatment, future research can focus on six core directions.

#### 6.2.1 Verification of molecular mechanisms and causal relationships

Existing studies confirm that EGCG regulates oxidative stress, neuroinflammation, autophagy, ferroptosis and NVU homeostasis, yet its direct molecular targets and hierarchical crosstalk among signaling pathways remain ambiguous. Future work should combine gene knockout, conditional knockout, cell-specific intervention, pharmacological blockade and single-cell multi-omics to clarify cell-specific effects of EGCG in neurons, microglia, astrocytes, cerebral endothelial cells and peripheral immune cells, and dissect causal links between direct target binding and downstream signaling alterations (Li et al., 2024; Kamboj et al., 2025).

#### 6.2.2 Clinically relevant stroke animal models

Subsequent *in vivo* research should prioritize aged, female stroke models and animals complicated with hypertension, diabetes, hyperlipidemia or atherosclerosis, combined with simulated intravenous thrombolysis or endovascular therapy to improve clinical extrapolability. Apart from acute infarct volume and short neurological scores, long-term endpoints including motor recovery, cognitive function, white matter integrity, brain network remodeling and quality of life should be incorporated for comprehensive evaluation of EGCG neuroprotection (Anderson and Song, 2024; Schmidt-Pogoda et al., 2024).

#### 6.2.3 Adjunct neuroprotective regimens combined with reperfusion therapy

As intravenous thrombolysis and mechanical thrombectomy become standard acute ischemic stroke care, EGCG is positioned as an adjunct agent post-recanalization rather than a substitute for reperfusion interventions. Future studies should identify the optimal intervention window (pre-reperfusion, intra-reperfusion, post-reperfusion) and systematically assess efficacy, safety, and hemorrhagic risk when EGCG is co-administered with rt-PA, thrombectomy, antiplatelets, anticoagulants, and lipid-lowering drugs, providing evidence for multi-modal comprehensive neuroprotective strategies (Powers et al., 2019; Greco et al., 2023; Anderson and Song, 2024; Schmidt-Pogoda et al., 2024; Bahr-Hosseini and Saver, 2025).

#### 6.2.4 Scalable brain-targeted delivery platforms

EGCG carrier design should balance brain penetration, biosafety, production reproducibility, and regulatory feasibility, prioritizing simple, scalable nanoformulations with well-defined compositions. Combined with ischemia-responsive release and BBB receptor-mediated targeting strategies to boost lesion accumulation. Apart from cerebral exposure detection, systematic long-term tissue distribution, metabolism, immunogenicity, and repeated-dose safety assessments are required to meet clinical translational demands (Ji et al., 2024; Lei et al., 2024).

#### 6.2.5 Construction of high-quality clinical evidence

Clinical trials should follow evidence-based medicine standards. Pharmacokinetic and safety trials in healthy volunteers and stroke convalescents should be conducted first to determine optimal dosing regimens, followed by dose-escalation studies and biomarker research in acute stroke patients. Ultimately, multicenter randomized controlled trials with functional recovery as primary endpoints are needed to systematically evaluate EGCG’s capacity to reduce disability and improve quality of life (Wang and You, 2017; Powers et al., 2019; Anderson and Song, 2024; Schmidt-Pogoda et al., 2024).

#### 6.2.6 Unified research and outcome evaluation standards

Uniform criteria should be established for EGCG purity, stability, administration routes, drug exposure measurement, nanoformulation quality control, and clinical efficacy assessment. Negative results and replication studies are encouraged to improve comparability and overall evidence quality across published literature. Standardized evaluation frameworks compliant with international regulatory standards should be formulated to provide reliable evidence for dosage optimization, regulatory approval and clinical promotion (Ji et al., 2024; Lei et al., 2024).

## 7 Conclusion

In summary, EGCG, the most abundant and bioactive natural polyphenol extracted from green tea, exerts multi-pharmacological effects including anti-oxidation, anti-inflammation, regulation of cell death and maintenance of NVU integrity. It elicits broad-spectrum neuroprotection *via* coordinated intervention in interconnected pathological cascades including oxidative stress, neuroinflammation, excitotoxicity, ferroptosis, BBB damage and NVU dysfunction. Recent advancements in nanotechnology have greatly improved EGCG stability, cerebral bioavailability and brain-targeting capacity, creating new opportunities for its clinical translation.

Nevertheless, current research on EGCG for stroke is dominated by cellular and animal experiments. Its clinical application is limited by insufficient oral bioavailability, poor BBB penetration, translational gaps between preclinical models and real patients, and a lack of high-quality human clinical trials. Key unresolved questions include standardized optimal dosage, administration route, therapeutic window, long-term safety, and synergistic benefits with recanalization therapies.

Overall, EGCG displays promising prospects in stroke neuroprotection due to its multi-target, multi-pathway regulatory characteristics. Future research should further clarify core molecular mechanisms, optimize brain-targeted delivery systems, establish unified quality and efficacy evaluation criteria, and launch well-designed multicenter randomized controlled trials to accelerate the translation of EGCG from laboratory research to clinical practice, delivering novel precision neuroprotective strategies for stroke patients.
